# MicroRNA-17 is downregulated in esophageal adenocarcinoma cancer stem-like cells and promotes a radioresistant phenotype

**DOI:** 10.18632/oncotarget.13940

**Published:** 2016-12-15

**Authors:** Niamh Lynam-Lennon, Susan Heavey, Gary Sommerville, Becky A.S. Bibby, Brendan Ffrench, Jennifer Quinn, Claudia Gasch, John J O’Leary, Michael F Gallagher, John V Reynolds, Stephen G Maher

**Affiliations:** ^1^ Trinity Translational Medicine Institute, Department of Surgery, Trinity College Dublin, St James's Hospital, Dublin 8, Ireland; ^2^ Cancer Biology and Therapeutics Lab, School of Life Sciences, University of Hull, Hull, United Kingdom; ^3^ Department of Histopathology, Trinity College Dublin, Sir Patrick Dun Laboratory, Central Pathology Laboratory, St James's Hospital, Dublin 8, Ireland; ^4^ Molecular Pathology Laboratory, Coombe Women and Infant's University Hospital, Dublin 8, Ireland

**Keywords:** esophageal adenocarcinoma, radioresistance, cancer stem-like cells, microRNA, predictive biomarker

## Abstract

Resistance to neoadjuvant chemoradiation therapy (CRT) remains a critical barrier to the effective treatment of esophageal adenocarcinoma (EAC). Cancer stem-like cells (CSCs) are a distinct subpopulation of cells implicated in the resistance of tumors to anti-cancer therapy. However, their role in the resistance of EAC to CRT is largely unknown. In this study, using a novel *in vitro* isogenic model of radioresistant EAC, we demonstrate that radioresistant EAC cells have enhanced tumorigenicity *in vivo*, increased expression of CSC-associated markers and enhanced holoclone forming ability. Further investigation identified a subpopulation of cells that are characterised by high aldehyde dehydrogenase (ALDH) activity, enhanced radioresistance and decreased expression of miR-17-5p. *In vitro*, miR-17-5p was demonstrated to significantly sensitise radioresistant cells to X-ray radiation and promoted the repression of genes with miR-17-5p binding sites, such as *C6orf120*. *In vivo*, miR-17-5p was significantly decreased, whilst *C6orf120* was significantly increased, in pre-treatment EAC tumour samples from patients who demonstrated a poor response to neoadjuvant CRT. This study sheds novel insights into the role of CSCs in the resistance of EAC to CRT and highlights miR-17-5p as a potential biomarker of CRT sensitivity and novel therapeutic target in treatment resistant EAC.

## INTRODUCTION

Esophageal cancer occurs worldwide and confers a dismal prognosis. In recent decades, there has been a dramatic epidemiological shift in the incidence of esophageal adenocarcinoma (EAC), with rates rising by 600% over the last 30 years [1]. EAC is now the predominant histological subtype in Europe and the United States. This increase is linked to key lifestyle factors, such as obesity, and incidence is expected to increase at a similar rate in the coming decades [2]. Despite improvements in surveillance and diagnosis, the overall cure rate for esophageal cancer is less than 17%, and 39% for localized disease, and results in over 400,000 estimated deaths globally each year [1].

Consequently, a multi-modal approach to treatment has been developed, with neoadjuvant chemoradiation therapy (CRT) followed by surgery increasingly becoming a standard of care in North America and Europe [3]. The tumor response to neoadjuvant CRT is currently the best predictor of overall and disease-free survival, with the attainment of a complete pathological response (pCR) associated with a five-year survival rate of up to 60% [4]. Whilst higher pCR rates are achieved with CRT than with induction chemotherapy alone [5, 6], unfortunately, approximately 70% of patients receiving neoadjuvant treatment show moderate or no response [7]. Consequently, this sub-group of patients are unnecessarily subjected to CRT-associated adverse events, including an increased risk of surgical complications [8, 9], as well as a delay to surgery, which can ultimately worsen prognosis and increase clinical expense [10]. Presently, standard clinicopathological factors do not predict treatment response and there are currently no predictive markers used routinely in the clinic. In addition, for those majority of EAC patients resistant to current standard CRT regimens, there are no alternative treatment strategies. Therefore, the need for both validated biomarkers predicting response to treatment and the development of novel treatment strategies to boost tumor responses to neoadjuvant CRT is currently unmet.

Current standard anti-cancer therapies, such as neoadjuvant CRT, have been developed and evaluated for their effectiveness in debulking the primary tumor. However, increasing evidence supports the hierarchical organization of cancers with a low frequency minority population of cancer cells with stem-like properties, currently termed cancer stem-like cells (CSCs). These CSCs are considered critical in the maintenance of tumors, and can be characterised predominantly by their ability to self-renew, repopulate a tumor following treatment with conventional cytotoxics and radiation and initiate and promote metastatic growth [11]. CSCs represent a distinct cell population, which have been clearly demonstrated to be a predominant cause of tumor resistance to CRT treatment [12]. Increasing evidence supports a role for CSCs in the pathogenesis and prognosis of esophageal squamous cell carcinoma [13–15], however, the role of CSCs in EAC is largely undetermined. The elucidation of CSC-related molecular pathways associated with resistance to CRT may identify both novel biomarkers predicting treatment response and novel therapeutic targets for improved treatment strategies in EAC. Increasing evidence suggests that the pro-tumorigenic properties of CSCs may result from epigenetic and post-transcriptional alterations, which alter multiple signalling pathways [16, 17]. MicroRNAs (miRNAs) are a family of novel non-coding RNA, which function to regulate gene expression [18]. MiRNA expression is intimately involved in tumorigenesis and cancer biology [18, 19] and evidence supports their role as regulators of the CSC phenotype [20]. We have previously demonstrated a role for miRNAs in the response of EAC to both X-ray radiation and chemotherapy [21–23], highlighting miRNAs as both potential predictive biomarkers of response and novel therapeutic targets to enhance the tumor response to treatment in EAC. However, the contribution of miRNAs in regulating CSC properties in EAC is largely unknown.

This study investigated the role of CSCs in the treatment resistance of EAC, utilising both novel *in vitro* models of radioresistant and chemoresistant EAC and tumour biopsies from EAC patients. The data demonstrate that radioresistant EAC cells have enhanced tumorigenicity *in vivo*, increased expression of CSC-associated markers and enhanced holoclone forming ability. Furthermore, this study identifies for the first time, a subpopulation of CSCs in EAC, which are characterised by increased ALDH activity, enhanced resistance to radiation and decreased expression of miR-17-5p. We also demonstrate for the first time that miR-17-5p functionally modulates sensitivity to radiation *in vitro* in EAC cells and alters expression of predicted miR-17-5p target genes, such as *C6orf120*. *In vivo*, miR-17-5p is significantly decreased, whilst target gene expression is significantly increased in pre-treatment tumour biopsies from patients who have a poor response to neoadjuvant CRT. Our data highlight a potential role for miR-17-5p as a predictive marker of response to neoadjuvant CRT and novel therapeutic target to enhance efficacy of CRT in EAC.

## RESULTS

### Radioresistant OE33 R cells demonstrate enhanced tumorigenicity in mice

To investigate the role of CSCs in the radioresistance of EAC, we utilised an isogenic cell line model of radioresistant EAC, previously generated in our laboratory [24]. The radioresistant OE33 R subline, which was generated by chronic irradiation with clinically-relevant fractionated doses of 2 Gy X-ray radiation, displays significantly enhanced resistance to radiation, when compared to its radiosensitive OE33 parent (OE33 P) cell line [24]. These two cell lines, of the same origin but with distinctly different radiosensitivities, provide a unique model with which to investigate the molecular determinants of response to radiation in EAC.

**Figure 1:**
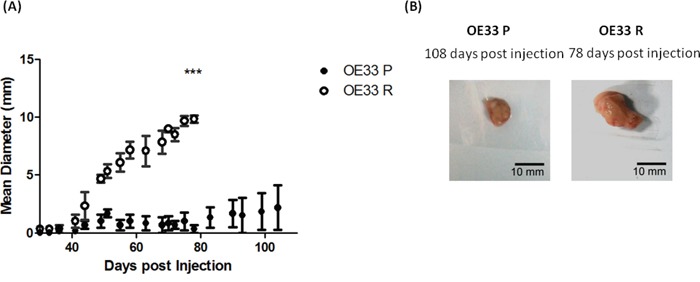
OE33 R cells demonstrate enhanced tumorigenicity *in vivo* **A**. OE33 P and OE33 R cells were implanted subcutaneously into NOD SCID mice (*n*=3). OE33 R cells demonstrated significantly enhanced tumorigenesis when compared to OE33 P cells. Data are presented as the mean ± SEM. Statistical analysis was performed using a two-tailed unpaired Student's t-test,***p < 0.0001 **B**. Representative images of tumors extracted from OE33 P and OE33 R cells injected into NOD SCID mice.

To investigate the tumorigenicity of this novel isogenic model *in vivo*, OE33 P and OE33 R cells were transplanted subcutaneously into the flank of immunocompromised NOD SCID mice. Mice were monitored weekly for tumor growth. OE33 R cells demonstrated higher tumorigenic potential; this manifested as higher graft success rates and corresponded to shorter latency and larger tumor volumes with respect to time, when compared to OE33 P cells (Figure 1A and 1B).

OE33 R cells produced palpable tumors in all animals transplanted (*n*=3) by day 30 post transplantation. By day 78 post transplantation, OE33 R cells had produced tumors with an average diameter of 9.8 mm. In contrast, OE33 P cells produced a palpable tumor in only one of the 3 transplanted animals, which at day 78 post transplantation had reached an average tumor diameter of 1 mm. As this isogenic model of radioresistance originates from the same OE33 cell line, the inherent genetic variation between OE33 P and OE33 R is reduced, when compared to using cell lines of different origin. Therefore, this may suggest that the enhanced tumorigenicity demonstrated by OE33 R cells is due to alterations in the biology of the population of cells, or a subpopulation of cells, such as CSCs, which have the ability to drive tumor growth, rather than alterations in specific gene/protein drivers of oncogenesis.

### Radioresistant OE33 R cells demonstrate enhanced ‘stemness’ properties

**Figure 2:**
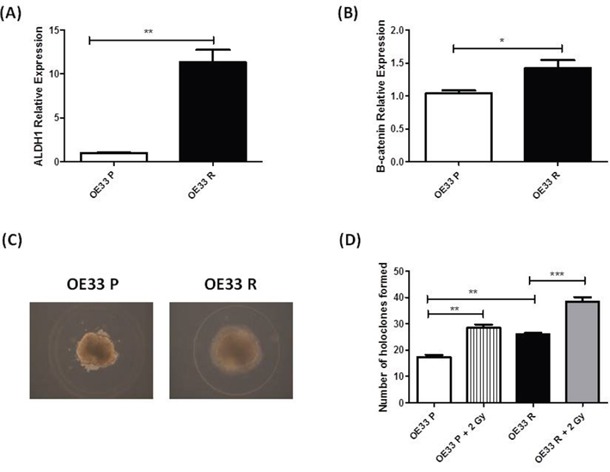
OE33 R cells demonstrate enhanced ‘stemness’ properties Expression of *ALDH1* and *β-catenin* was assessed in OE33 P and OE33 R cells by qPCR. Expression levels of **A**. *ALDH1* and **B**. *β-catenin* were significantly increased in OE33 R cells, when compared to OE33 P cells **C**. OE33 P and OE33 R cells were plated under high salt agar conditions. Representative images of resulting holoclones (Magnification 10 ×) **D**. OE33 R cells have significantly enhanced holoclone forming capacity both basally and following irradiation with 2 Gy, when compared to OE33 P cells. Data are presented as the mean ± SEM from 3 independent experiments. Statistical analysis was performed using an ANOVA with Tukey *post hoc* test, **p< 0.05, ***p< 0.005.

To investigate if the enhanced tumorigenicity demonstrated by OE33 R cells *in vivo* may indeed be due to an enriched CSC population, we assessed several ‘stemness’ properties in OE33 R and OE33 P cells. Expression of the putative CSC markers *ALDH1* and *β-catenin*, which are implicated in the regulation of CSCs [25], was investigated in OE33 P and OE33 R cells by qPCR. Expression of both *ALDH1* and *β-catenin* was significantly increased in OE33 R cells, when compared to OE33 P (Figure 2A and Figure 2B), suggesting an enrichment of CSCs in the OE33 R cell line. To further investigate this, the holoclone forming ability of OE33 P and OE33 R cells was assessed at basal level and following irradiation with 2 Gy X-ray radiation. Holoclones are colonies with distinct morphology, which are capable of extensive proliferation and self-renewal and are demonstrated to be enriched for CSCs [26]. Both OE33 P and OE33 R cells formed holoclones, which displayed characteristic morphology (Figure 2C). However, the potential for holoclone formation was significantly higher in radioresistant OE33 R cells, when compared to radiosensitive OE33 P cells, both basally and following irradiation with 2 Gy (Figure 2D). Together, these data suggest that OE33 R cells are enriched for CSCs, which may be an important feature underlying their enhanced tumorigenicity *in vivo* and resistance to X-ray radiation *in vitro*.

### Radioresistant and chemoresistant EAC cells are enriched for an ALDH+ve cell population

**Figure 3:**
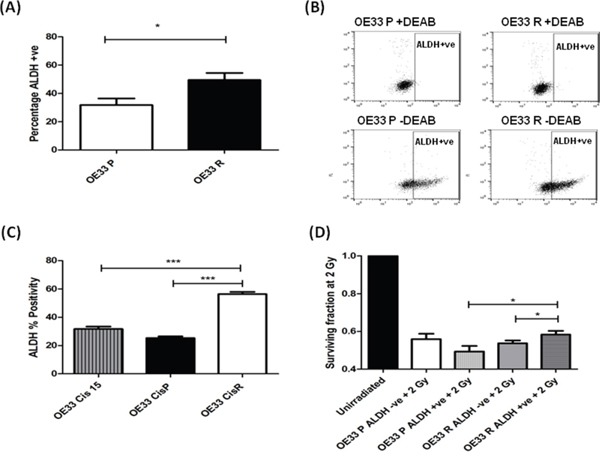
Radioresistant and cisplatin-resistant EAC cells have increased ALDH enzymatic activity, and is associated with a radioresistant phenotype **A**. OE33 P and OE33 R cells were stained for ALDH and fluorescence was assessed using a MoFlow cell sorter. As a negative control, cells were treated with the specific ALDH inhibitor DEAB. OE33 R cells demonstrate significantly higher levels of ALDH+ve cells, when compared to OE33 P cells. Data are presented as the mean ± SEM from 7 independent experiments. Statistical analysis was performed using an unpaired two-tailed Student's *t*-test, *p < 0.05 **B**. Representative flow images of ALDH staining in OE33 P and OE33 R cells. DEAB served as a negative control. **C**. ALDH activity was assessed in an isogenic model of EAC cisplatin-resistance. Cisplatin-resistant cells OE33 CisR demonstrated significantly higher levels of ALDH+ve cells, when compared to cisplatin-sensitive OE33 CisP and OE33 Cis15 cells. Data are presented as the mean ± SEM from 3 independent experiments. Statistical analysis was performed using an unpaired two-tailed Student's *t*-test, ***p < 0.001 **D**. ALDH-ve and ALDH +ve populations from OE33 P and OE33 R cells were sorted using a MoFlow cell sorter and radiosensitivity to 2 Gy X-ray radiation was assessed using clonogenic assay. OE33 R ALDH+ve populations demonstrate significantly enhanced survival to radiation at 2 Gy, when compared to OE33 R ALDH-ve cells and OE33 P ALDH+ve cells. Data are presented as the mean ± SEM from 7 independent experiments. Statistical analysis was performed using a paired and unpaired two-tailed Student's *t*-test, respectively, *p < 0.05.

Given the significantly increased expression of *ALDH1* mRNA in OE33 R cells (Figure 2A), we assessed if ALDH activity was also increased in OE33 R cells, using flow cytometry. Supporting the alterations at mRNA level, radioresistant OE33 R had a significantly higher percentage of ALDH+ve cells (49%), when compared to radiosensitive OE33 P (32%) (Figure 3A and 3B), again suggesting that OE33 R cells are enriched for CSCs. To investigate if this increase in ALDH+ve cells was specific to radioresistant cells, we also assessed ALDH activity in an isogenic EAC cell line model of cisplatin resistance. To generate this isogenic model of cisplatin resistance, OE33 cells were treated with 1 μM cisplatin until the emergence of a cisplatin resistant sub-line, termed OE33 CisR, having received 21 cycles of cisplatin treatment. OE33 CisR cells demonstrated a significant increase in surviving fraction following treatment with 1 μM cisplatin, when compared to their vehicle controlled age- and passage-matched cisplatin sensitive counterpart, termed OE33 CisP (Supplementary Figure 1). Interestingly, OE33 CisR cells demonstrated a significantly higher percentage of ALDH+ve cells (56%), when compared to OE33 CisP cells (25%) (Figure 3C), supporting a role for ALDH+ve CSCs in the resistance of these cells to cisplatin. Importantly, when ALDH activity was assessed in OE33 cells that received only 15 cycles of cisplatin treatment (OE33 Cis15), and do not display resistance to cisplatin compared with control (data not shown), there was no significant increase in ALDH activity (Figure 3C), further supporting a role for ALDH activity in the acquired resistance to cisplatin in EAC cells. Taken together, these data suggest that enrichment of ALDH+ve CSCs is associated with resistance to X-ray radiation and cisplatin in EAC cells.

### OE33 R ALDH+ve cells demonstrate enhanced radioresistance compared with OE33 R ALDH-ve cells

To investigate if the ALDH+ve population may be involved in the radioresistance phenotype displayed by OE33 R cells, ALDH+ve and ALDH-ve populations from both OE33 P and OE33 R cells were separated by fluorescence-activated cell sorting and comparative radiosensitivity was assessed using the gold standard clonogenic assay. ALDH+ve populations isolated from OE33 R cells were demonstrated to be significantly more resistant to radiation at a clinically-relevant dose of 2 Gy, when compared to the ALDH-ve populations (Figure 3D), suggesting that this minority subpopulation may be the effectors of the enhanced radioresistance of the OE33 R cell line. Interestingly, ALDH+ve cells from OE33 R cells also demonstrated significantly higher survival following 2 Gy, when compared to ALDH+ve populations from OE33 P cells (Figure 3D), suggesting that the ALDH+ve population in OE33 R cells has additional molecular alterations that promote enhanced resistance to radiation.

### miR-17-5p is decreased in radioresistant OE33 R ALDH+ve populations

**Figure 4:**
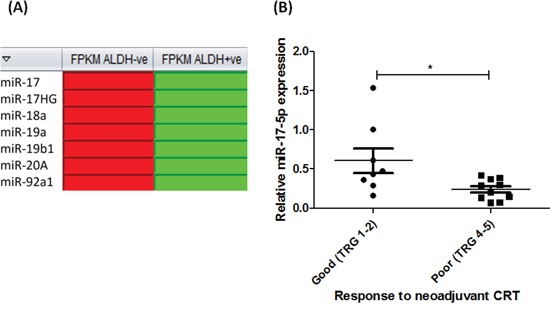
miR-17-5p is decreased in radioresistant EAC cells *in vitro* and *in vivo* **A**. Digital gene expression analysis, demonstrated as heat maps, of miR-17~92 family members significantly altered in OE33 R ALDH+ve populations, when compared to OE33 R ALDH-ve populations (red and green boxes indicate upregulation and downregulation, respectively) **B**. miR-17-5p expression is significantly decreased in pre-treatment diagnostic tumor biopsies from EAC patients with a poor response to neoadjuvant CRT (TRG 4 and 5), when compared to good responders (TRG 1 and 2) (*n*=18) [23]. Statistical analysis was performed using an unpaired, 2-tailed Student's *t*-test, *p < 0.05.

To investigate potential molecular mechanisms underlying the enhanced radioresistance of OE33 R ALDH+ve cells, digital gene expression profiling of OE33 R ALDH+ve and OE33 R ALDH-ve cells was performed. Interestingly, 8 miRNAs were identified as being significantly altered between relatively radiosensitive ALDH-ve and radioresistant ALDH+ve populations from OE33 R, supporting a role for miRNAs in the regulation of these distinct cellular subpopulations. Of particular interest, several members of the miR-17~92 cluster (miR-17-5p, miR-17HG, miR-18a, miR-19a, miR-19b1, miR-20A and miR-92a1) (Figure 4A), were demonstrated to be significantly decreased in radioresistant OE33 R ALDH+ve cells, when compared to OE33 R ALDH-ve cells (Fragments per kilobase of exon per million fragments mapped (FPKM) of 4.76 versus 57.10, respectively). We have previously demonstrated one of these miRNAs, miR-17-5p, to be significantly decreased in pre-treatment diagnostic tumor biopsies from EAC patients (patient characteristics outlined in Table 1) who subsequently have a poor response to neoadjuvant CRT [23] (Figure 4B), supporting a role for miR-17-5p in the tumor response to treatment. Taken together, these data suggest that the downregulation of miR-17-5p in radioresistant OE33R ALDH+ve cells, may provide a mechanism underlying their resistance to radiation.

**Table 1:** Patient characteristics

	miR-17-5p Study(*n* = 18)	Gene Target Study(*n* = 30)
Male	16	25
Female	2	5
Age (years)^a^	63 (37-75)	61 (37-75)
**Clinical TNM Stage**		
I	0	1
IIa	6	8
IIb	2	4
III	10	17
IV	0	0
**TRG**		
1	3	5
2	5	6
3	0	9
4	8	8
5	2	2

Abbreviations: ^a^Values given are mean (range); TNM, Tumor-node-metastasis clinical staging classification; TRG, Tumour regression grade.

### miR-17-5p overexpression sensitises radioresistant EAC cells to radiation and results in repression of predicted gene targets

**Figure 5:**
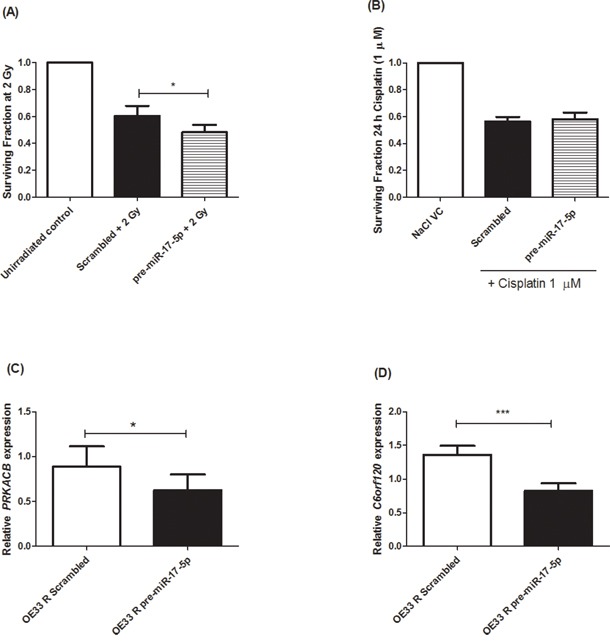
miR-17-5p sensitises radioresistant OE33 R cells to radiation and downregulates *PRKACB* and *C6orf120* expression **A**. miR-17-5p was transiently overexpressed in OE33 R cells. miR-17-5p significantly sensitised OE33 R cells to 2 Gy radiation, when compared to a scrambled control. **B**. miR-17-5p does not alter sensitivity to cisplatin (1 μM) relative to a scrambled control. Overexpression of miR-17-5p resulted in significant downregulation of **C**. *PRKACB* and **D**. *C6orf120*, when compared to a scrambled control. Data are presented as the mean ± SEM from 3 independent experiments. Statistical analysis was performed using a paired Student's *t*-test, *p < 0.05; ***p < 0.001.

The significant decrease in miR-17-5p expression in radioresistant OE33 R ALDH+ve cells and tumors from EAC patients resistant to CRT [23], suggests that downregulation of miR-17-5p may provide a mechanism supporting radioresistance in EAC. To investigate if miR-17-5p plays a functional role in modulating radiosensitivity, transient overexpression of miR-17-5p was performed in radioresistant OE33 R cells. Overexpression of miR-17-5p in transfected cells was confirmed by qPCR (Supplementary Figure 2). OE33 R cells overexpressing miR-17-5p were demonstrated to be significantly more sensitive to a clinically-relevant dose of 2 Gy X-ray radiation, when compared to a scrambled non-targeting control (Figure 5A), thus supporting a functional role for miR-17-5p in the response of EAC to X-ray radiation and further supporting downregulation as a mechanism for radioresistance in OE33 R ALDH+ve cells and poor responder tumors.

To investigate if this sensitising effect was specific to X-ray radiation, transient overexpression of miR-17-5p was performed in cisplatin-resistant OE33 CisR cells and the sensitivity to cisplatin (1 μM) was assessed. OE33 CisR cells overexpressing miR-17-5p did not demonstrate any significantly altered sensitivity to cisplatin (Figure 5B), suggesting that, *in vitro*, miR-17-5p does not play a functional role in modulating the response to cisplatin in EAC cells. To elucidate potential gene targets of miR-17-5p, the Ingenuity Pathway Analysis (IPA) program was used to identify gene targets with predicted miR-17-5p binding sites. *PRKACB* and *C6orf120* were two genes identified to have highly predicted miR-17-5p binding sites, suggesting the potential post-transcriptional regulation of these genes by miR-17-5p. Expression of *PRKACB* (Figure 5C) and *C6orf120* (Figure 5D) was assessed in cells overexpressing miR-17-5p and both genes were demonstrated to be significantly decreased in OE33 R cells overexpressing miR-17-5p, when compared to a scrambled non-targeting control, supporting miR-17-5p-mediated negative regulation of gene targets.

### C6orf120 is significantly increased in EAC tumors resistant to neoadjuvant CRT

**Figure 6:**
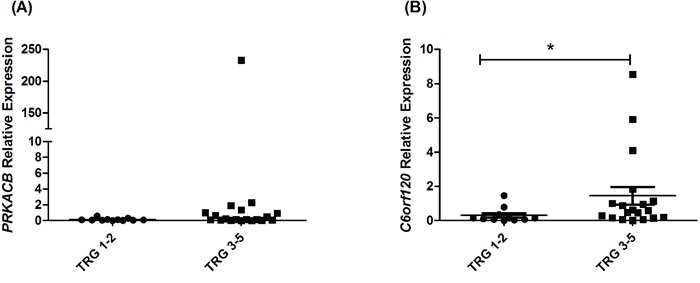
*C6orf120* is significantly increased in patients having a poor response to neoadjuvant CRT Expression of **A**. *PRKACB* and **B**. *C6orf120* was assessed by qPCR in pre-treatment EAC tumor biopsies from patients (*n* = 30) who subsequently received neoadjuvant CRT. Statistical analysis was performed using a Mann Whitney U test, *p < 0.02.

Given that miR-17-5p expression is significantly lower in pre-treatment tumor biopsies from EAC patients who subsequently have a poor response to neoadjuvant CRT ([23] and Figure 4A), we hypothesised that *C6orf120* and *PRKACB* would consequently be upregulated in poor responder tumors. The expression of *C6orf120* and *PRKACB* was assessed by qPCR in pre-treatment tumor biopsies from EAC patients (*n*=30), who subsequently received neoadjuvant CRT. Of the 30 patients, 37% were classified as good responders (TRG 1 and 2), whilst 63% were classified as poor responders (TRG 3, 4 and 5). Again, patient characteristics are outlined in Table 1. *PRKACB* demonstrated a trend towards increased expression in poor responders (*p* < 0.10), when compared to good responders, while, this was not statistically significant (Figure 6A). However, *C6orf120* expression was demonstrated to be significantly increased in tumor biopsies from patients who had a poor response to neoadjuvant CRT, when compared to good responders (Figure 6B), suggesting that the decreased expression of miR-17-5p in poor responder tumors may provide a mechanism for increased *C6orf120* expression *in vivo*.

## DISCUSSION

The identification of biomarkers predicting therapeutic response and novel therapeutic targets to enhance response to neoadjuvant CRT in EAC is crucial to improving treatment efficacy and survival for patients. CSCs are identified as a distinct tumor cell population, implicated in playing a causative role in inherent tumor resistance to conventional anti-cancer treatments, such as chemotherapy and radiation therapy [12], and in accelerated repopulation and acquired resistance post treatment, highlighting their potential as novel predictors of response and a putative therapeutic target. However, the role of CSCs in the resistance of EAC to treatment is largely unknown.

In this study, we utilised an isogenic model of radioresistant EAC previously generated in our laboratory [24] to investigate the potential role of CSCs in the resistance to radiation treatment in EAC. Radioresistant OE33 R cells were demonstrated to have enhanced tumorigenicity in mice, increased expression of CSC-associated markers and enhanced holoclone forming potential when compared to radiosensitive OE33 P cells, supporting a role for CSCs in this radioresistant EAC cell line. This supports several previous studies, which have highlighted a role for CSCs in the pathogenesis of EAC [27–29].

The identification and isolation of CSCs can present a challenge [30]. ALDH activity has been successfully used as a CSC marker in a number of solid tumors, including esophageal [27, 29], breast [31], lung [32], and prostate [33]. Here, we demonstrate that ALDH activity correlates with acquired resistance to both X-ray radiation and cisplatin *in vitro* in EAC cells, supporting a role for this ALDH positive (ALDH+ve) subpopulation in the resistance of EAC cells to both cytotoxic treatments. Furthermore, functional analysis of these distinct ALDH negative (ALDH-ve) and ALDH+ve subpopulations from both radioresistant and radiosensitive cells, demonstrated they had different relative radiosensitivities. Notably, the minority ALDH+ve subpopulation of cells isolated from the radioresistant OE33 R cell line were significantly more resistant to a clinically-relevant dose of 2 Gy radiation, when compared to the majority ALDH-ve subpopulation in OE33 R, suggesting this population as a predominant underlying cause of radioresistance in these cells. This supports two recent studies in prostate [34] and head and neck cancer [35], which demonstrated that cells with high ALDH activity have enhanced resistance to radiation and maintain their tumorigenic properties, when compared to ALDH-ve cells. Furthermore, a recent study by Ajani and colleagues demonstrated that high ALDH1 expression in pre-treatment EAC biopsies is associated with subsequent resistance to neoadjuvant CRT and is increased *in vitro* in chemoresistant EAC cells [29]. However, this study is the first to demonstrate the enhanced radioresistance of ALDH+ve cells in EAC. In this study we also demonstrate that ALDH+ve cells isolated from the radioresistant OE33 R line are significantly more resistant to 2 Gy radiation, when compared to ALDH+ve populations from radiosensitive OE33 P cells. This may suggest that the ALDH+ve population in OE33 R cells have undergone additional molecular alterations that result in enhanced resistance to radiation, and that the ALDH+ve population can potentially be further segregated into distinct sub-subpopulations. This is supported by a previous study in pancreatic cancer, which demonstrated that CSC populations derived from patient-derived xenograft (PDX) models are heterogenous, with subpopulations of CSCs demonstrating different quiescent and chemoresistance profiles [36]. The fundamental mechanisms of chemotherapy and radiotherapy resistance are likely to be polygenic in nature. Therefore, it must be considered that additional mutational and genetic events are also likely involved in the resistance of these isogenic cell line models of radioresistant and chemoresistant EAC.

Several studies have demonstrated that whilst CSCs and their non-tumorigenic progenies often share a common genotype, they display altered epigenetic profiles, which can result in altered phenotypes [20, 37, 38]. This suggests that molecular reprogramming could be a fundamental mechanism underpinning the observed enhancement of radioresistance in the OE33 R ALDH+ve cells. Supporting this, 8 miRNAs were demonstrated to be significantly altered between ALDH+ve and ALDH-ve populations from OE33 R, suggesting a role for miRNAs in the enhanced radioresistance of the OE33 R ALDH+ve population. Interestingly, several members of the miR-17~92 family (miR-17-5p, miR-17HG, miR-18a, miR-19a, miR-19b1, miR-20A and miR-92a1) were demonstrated to be significantly decreased in radioresistant OE33 R ALDH+ve cells, when compared to radiosensitive OE33 R ALDH-ve populations. The miR-17~92 cluster is located on chromosome 13q31, and is transcribed as a single primary transcript, which is processed to produce distinct, individual miR-17~92 members [39]. Evidence suggests a dual role for the miR-17~92 cluster in tumorigenesis, with evidence to support both oncogenic [40, 41] and tumor suppressive [42, 43] roles, suggesting a tumor/tissue-specific role for these miRNAs. Interestingly, we have previously demonstrated that one of these altered miRNAs, miR-17-5p, is significantly decreased in pre-treatment EAC tumor biopsies from patients who subsequently have a poor response to neoadjuvant CRT [23], supporting loss of miR-17-5p as a potential driver mechanism of resistance to radiation both *in vitro* and *in vivo*. Supporting this, miR-17-5p was demonstrated to play a functional role in modulating sensitivity to radiation, significantly sensitising radioresistant OE33 R cells to 2 Gy X-ray radiation *in vitro*. miR-17-5p overexpression did not alter sensitivity to cisplatin, suggesting that miR-17-5p may play a predominant role in the response to radiation. This suggests that whilst ALDH activity may be a general CSC marker, commonly applicable to both cisplatin- and radiation-resistant cells, miR-17-5p expression might further delineate the CSC populations, with downregulation of miR-17-5p specific to a radioresistant phenotype. As EAC biopsies are taken routinely at diagnosis, this suggests that expression of miR-17-5p could potentially be incorporated into a ‘companion diagnostic’ prior to initiation of neoadjuvant CRT to aid in the identification of the most responsive patient subgroup, allowing for a more personalized approach to treatment. However, further validation of miR-17-5p as a predictive marker of CRT response in a larger patient cohort is required.

Our data suggest that in EAC tumors, downregulation of miR-17-5p provides a mechanism for resistance to neoadjuvant radiation and chemotherapy. This is supported by a study in esophageal squamous cell carcinoma, which demonstrated downregulation of miR-17 in a model of acquired radioresistance [44]. In addition, a recent study in breast cancer demonstrated that overexpression of miR-17 enhances sensitivity to UV radiation and chemotherapy [45]. Furthermore, in pancreatic cancer, expression of the miR-17~92 cluster is decreased in chemoresistant CSCs and overexpression enhances sensitivity to gemcitabine [36]. Consistent with our results, the region encoding miR-17 has been demonstrated to undergo loss of heterozygosity in a number of tumor types [46, 47]. Moreover, several studies have highlighted a role for mutations and genomic rearrangements in the pathogenesis of EAC [48, 49], with both losses and gains at 13q highlighted [50, 51], suggesting that genomic alterations may provide a mechanism for the decreased miR-17-5p expression demonstrated here in tumors of poorly responding patients.

Interestingly, miR-17-5p overexpression resulted in a downregulation of *PRKACB* and *C6orf120*, which are predicted to have binding sites for miR-17-5p, supporting miR-17-5p-mediated negative gene regulation. Supporting our *in vitro* results, *C6orf120* was demonstrated to be significantly increased in tumors from poor responders. *C6orf120* encodes an N-glycosylated protein, of which the function is largely unknown. A previous study demonstrated that C6orf120 is secreted from HepG2 liver cancer cells via the classical ER-Golgi secretory pathway [52]. Furthermore, C6orf120 was demonstrated to induce endoplasmic reticulum stress-associated apoptosis in CD4^+^ T cells, but not CD8^+^ T cells, suggesting an immunoregulatory function for this protein [52]. It is now well established that the immune system plays an important role in the tumor response to radiation, with radiation therapy augmenting the anti-tumor immune response [53, 54]. Whilst traditionally anti-tumor immune responses have been largely attributed to CD8^+^ T cells, increasing evidence supports a role for CD4^+^ T cells in the anti-tumor response [55, 56]. Supporting this, activated CD4^+^ T cells have been demonstrated to sensitise cervical cancer and glioma cells to the effects of γ-radiation *in vitro*, supporting a role for CD4^+^ T cells in the tumor response to radiation [57]. Taken together, our data may suggest that EAC tumors demonstrating decreased miR-17-5p expression and concomitantly increased C6orf120 expression will have a poor response to radiation via C6orf120-mediated immunosuppression of CD4^+^ T cells. However, further work is required to fully elucidate the mechanism of this resistance to neoadjuvant CRT.

This study provides evidence that a subpopulation of cells with CSC properties exists in EAC, which are characterised by increased ALDH activity, enhanced resistance to radiation and decreased expression of miR-17-5p. For the first time, we demonstrate that miR-17-5p is a functional modulator of radioresistance *in vitro*, and is decreased in EAC tumours from patients who have a poor response to neoadjuvant CRT. This study highlights for the first time a potential role for miR-17-5p as a biomarker predicting response to neoadjuvant CRT in EAC, which would facilitate improved patient stratification, as well as a novel therapeutic target to boost the efficacy of CRT in EAC.

## MATERIALS AND METHODS

### Ethics statement

Investigation has been conducted in accordance with the ethical standards according to the Declaration of Helsinki and according to national and international guidelines and has been approved by the authors' institutional review board.

### Cells lines and cell culture

The human EAC line OE33 was obtained from the European collection of cell cultures. OE33 P and OE33 R cells were generated and cultured in our laboratory as previously described [24]. Briefly, OE33 cells were chronically irradiated with 2 Gy X-ray radiation every ~7-10 days, until a radioresistant subline (OE33 R) was generated, having received a cumulative dose of 50 Gy. Radiosensitivity was assessed by clonogenic assay. Parental cells (OE33 P) were mock irradiated. The cisplatin resistant variant of the OE33 cell line (OE33 CisR) was derived from the original parent cell line (OE33). Cells were treated with 1 μM cisplatin or PBS (NaCl) vehicle control for 72 h. Treatment was then discarded and replaced with complete media for 72 h, and this cyclic treatment regimen was repeated for approximately 6-months. During this time, cells were subcultured at 80-90% confluence and cisplatin sensitivity was assessed throughout the 6-month period via clonogenic assay.

### Animal experiments

All animal experiments were approved by the Ethics Board of Trinity College Dublin and licensed by the Health Products Regulatory Authority. Experiments were carried out in line with the United Kingdom Co-ordinating Committee on Cancer Research (UKCCCR) guidelines. Female 7-9 week old NOD SCID (NOD.CB17-*Prkdc^scid^*/NCrHsd) mice were obtained from Harlan Laboratories. Mice were subcutaneously injected, above the right hind-limb, with 1 × 10^3^ cells (either OE33 P or OE33 R) in a 100 μl volume of 20% high concentration Matrigel (Corning) and 80% Ham's F12 media (Lonza). Mice were monitored 2-3 times per week for indications of tumor development and tumor growth was measured using calipers. Tumors were measured on 2 perpendicular axes, to account for irregular tumour dimensions and the mean of these measurements was used to calculate the mean tumor diameter. Animals were sacrificed when tumors reached a mean tumor diameter of 10 mm, and individual replicates varied slightly in time taken to reach the 10 mm end-point. Growth of OE33 R tumors was terminated at 78 days post-injection, whilst OE33 P tumor growth was terminated at 108 days post-injection, coinciding with the maximum mean tumor diameter limit.

### RNA isolation

Total RNA was isolated from cells using a miRNeasy Mini Kit (Qiagen) as per the manufacturer's instructions. Total RNA and miRNAs were isolated from patient samples using an All-in-One purification kit (Norgen Biotek), as per the manufacturer's instructions. RNA was quantified using a Nanodrop 1000 spectrophotometer v3.3 (Thermo Scientific).

### Quantitative PCR

For assessment of gene expression in cell lines and patient samples, total RNA was reverse transcribed to cDNA using a High Capacity cDNA Reverse Transcription Kit (Thermo Fisher Scientific) or random hexamers (Invitrogen) and Bioscript enzyme (Bioline), respectively. qPCR was performed using a TaqMan® assay kit (Thermo Fisher Scientific) and 18S was used as an endogenous control for data normalization. qPCR was performed using an ABI Prism 7900HT real-time thermal cycler or QuantStudio 5 PCR system (Thermo Fisher Scientific). qPCR data were analysed by the 2^-ΔΔCt^ (Livak) method [58].

### Holoclone generation

Holoclones were generated as previously described [59]. Briefly, a sterile solution of agarose (1% w/v) and sodium chloride (1% w/v) was added to 10 cm petri dishes (10 mL/dish) and allowed to set at room temperature for 20 min. Petri dishes were supplemented with complete RPMI-1640 media (20 mL). Cells were seeded at a density of 1 × 10^6^ cells/dish and were maintained at 37°C in 95% humidified air containing 5% CO_2_. Resulting holoclones were examined microscopically to confirm holoclone morphology [59].

### Aldehyde dehydrogenase assay and cell sorting

Aldehyde dehydrogenase (ALDH) enzyme activity was assessed using the Aldefluor® assay (Stem Cell Technologies), according to the manufacturer's instructions. Briefly, cells were trypsinised and resuspended at a density of 1 × 10^6^ cells/mL in Aldefluor® assay buffer containing ALDH substrate (bodipy-aminoacetaldehyde) (5 μL/mL). Immediately following this, half of the resuspended cells were added to a tube containing the ALDH inhibitor diethylaminobenzaldehyde (DEAB), to provide a negative control. All samples were incubated for 45 min at 37°C with mild agitation. Cells were pelleted by centrifugation, resuspended in Aldefluor® assay buffer and analysed using a CyAn flow cytometer (Beckman Coulter). For cell sorting assays, cells were filtered using cell strainers (70 μm) and sorted into Aldefluor positive (ALDH+ve) and Aldefluor negative (ALDH-ve) cells using a MoFlow cell sorter (Beckman Coulter).

### Irradiation

Irradiation was performed using a Gulmay Medical RS 225 X-ray generator (Gulmay Medical), at a dose rate of 3.25 Gy per min.

### Clonogenic assay

For ALDH-associated irradiation experiments, ALDH+ve and ALDH-ve sorted cell populations were seeded at optimized clonogenic cell seeding densities (1.5 × 10^3^ and 3 × 10^3^ cells/well for control and irradiated cells, respectively) in 6-well plates. Cells were allowed to adhere overnight and were irradiated with 2 Gy X-ray radiation, whilst controls were mock-irradiated. For miR-17-5p overexpression irradiation experiments, transfected cells were irradiated with 2 Gy X-ray radiation, whilst controls were mock-irradiated, at 24 h post transfection and seeded at optimised clonogenic densities (as described above) in 6-well plates at 1 h post irradiation. For miR-17-5p overexpression cisplatin experiments, cells were seeded at optimised clonogenic densities (as described above) in 6-well plates at 24 h post transfection and allowed to adhere for 24 h. Cells were treated with 1 μM cisplatin or NaCl vehicle control and the medium replaced 24 h post treatment.

For all clonogenics experiments, cells were then incubated at 37°C in 5% CO_2_/95% air for 8-14 days to allow colonies to form. Colonies were fixed and stained with crystal violet (70% methanol, 30% H_2_O, 0.1% w/v crystal violet) for 1 h at room temperature, followed by destaining in H_2_O. Air-dried plates were imaged and colonies were counted using a Colcount instrument (Oxford Optronics). The surviving fraction was calculated as previously described [24].

### Digital gene expression analysis

Total RNA was extracted from OE33 R ALDH-ve and OE33 R ALDH+ve sorted cells and 1.5 μg prepared for shipping as advised by LC Sciences, Texas, USA. LC Sciences performed whole transcriptome digital RNA-seq using Illumina sequencing by synthesis technology. Briefly, for sequencing library preparation 1.5 μg total RNA was purified for poly-A containing mRNA molecules using poly-T oligo-attached magnetic beads from the mRNA-Seq sample preparation kit. Subsequently mRNA was fragmented, phosphatase and PNK treated, and 3’ and 5’ adapters ligated to the RNAs in preparation for cDNA synthesis. Following reverse transcription and amplification to selectively enrich fragments with 3’ and 5’ adapters, samples underwent DNA fragment enrichment via first and second PCR clean up. Resultant sample libraries were quality controlled using the Agilent 2100 bioanalyser. Sequencing was performed on a HiSeq 2500 platform using 50 bp SE cycle runs. For data processing, after removing the 3’ adapter (TGGAATTCTCGGGTGCCAAGG), reads with a length of less than 10 nt were excluded. The remaining reads were considered mappable reads. Mappable reads were aligned to genome using Tophat_v2.0.13. For pairwise differential gene expression analysis abundance was normalised and evaluated in FPKM using the Cuffdiff module of Cufflinks_v2.2.1. For gene ontology (GO) analysis of genes demonstrating a significant difference a q-value representing a false discovery rate adjusted p-value of <0.05 was employed. Analysed data sets including GO and KEGG analysis were provided by LC Sciences.

### miR-17-5p transfection

Transient overexpression of miR-17-5p was performed by reverse transfection of Pre-miR-17-5p or scrambled non-targeting control miRNA precursor molecules (5 nM) (Ambion), using Lipofectamine 2000 transfection reagent (Invitrogen), as per the manufacturer's instructions. Cells were transfected at a seeding density of 2.4 × 10^5^ cells/well in a 6-well plate.

### Patients, treatment and histology

Following ethical approval (Joint St James's Hospital/AMNCH ethical review board) and written informed consent, diagnostic biopsy specimens were obtained from patients with a diagnosis of operable EAC, prior to neoadjuvant therapy. All patients included in the study received a complete course of neoadjuvant CRT. Chemotherapy consisted of 2 courses of 5-fluorouracil (5-FU) and cisplatin, as previously described [60]. Patients received 40.05 Gy in 15 daily fractions (2.67 Gy/fraction) over 3-weeks as previously described [60]. Surgical resection involved transthoracic esophagectomy, including en-bloc lymphadenectomy of the abdominal and mediastinal nodes, and was performed approximately 1-month following completion of the CRT regimen. All resected oesophagectomy specimens were routinely assessed by an experienced pathologist. Tumor response to treatment was assigned 1 of 5 tumor regression grades (TRG), TRG 1 (complete regression) to TRG 5 (no regression) based on the presence of residual cancer cells and the degree of fibrotic change, as previously described [61]. Responders were classified as patients achieving a TRG of 1 or 2, whilst non-responders were classified as patients having a TRG of 3, 4 or 5, as previously described [22].

### Tissue collection

Diagnostic endoscopic biopsies were obtained by a qualified endoscopist prior to neoadjuvant therapy. Immediately adjacent tissue was taken for histologic confirmation, which was performed using routine hematoxylin and eosin staining. Specimens were immediately placed in RNA later (Ambion) and refrigerated for 24 h, before removal of RNA later and storage in a designated institutional biorepository at -80°C.

### Statistical analysis

Statistical analysis was carried out using GraphPad InStat v3 (GraphPad software Inc). All data are expressed as mean ± the standard error of the mean (SEM). Statistical analysis was performed using a 2-tailed Student's *t*-test, one-way ANOVA with Tukey *post-hoc* test or Mann-Whitney U non-parametrical testing. For all statistical analyses, differences were considered to be statistically significant at *p* ≤ 0.05.

## SUPPLEMENTARY MATERIALS FIGURES AND TABLES


