# Supplementary material for: MicroRNA-17 is downregulated in esophageal adenocarcinoma cancer stem-like cells and promotes a radioresistant phenotype
Source: Oncotarget. 2016 Dec 15;8(7):11400–13. doi: 10.18632/oncotarget.13940 (PMC5355274; doi:10.18632/oncotarget.13940)
Supplement: Supplementary file 1 [file oncotarget-08-11400-s001.pdf]

MicroRNA-17 is downregulated in esophageal adenocarcinoma cancer stem-like cells and promotes a radioresistant phenotype

SUPPLEMENTARY MATERIALS AND METHODS

Quantitative PCR

miR-17-5p overexpression was confirmed in OE33 R transfected cells at 24 h post transfection. Total RNA (10 ng) was reverse transcribed to cDNA using a TaqMan® MicroRNA Reverse Transcription Kit (Thermo Fisher Scientific), as per the manufacturer's instructions. qPCR was performed using a TaqMan® miRNA assay kit (Thermo Fisher Scientific) and RNU6B was used as an endogenous control for data normalization. qPCR was performed using an ABI Prism 7900HT real-time thermal cycler (Thermo Fisher Scientific). qPCR data were analysed by the $2^{-\Delta\Delta Ct}$ method.

SUPPLEMENTARY FIGURES

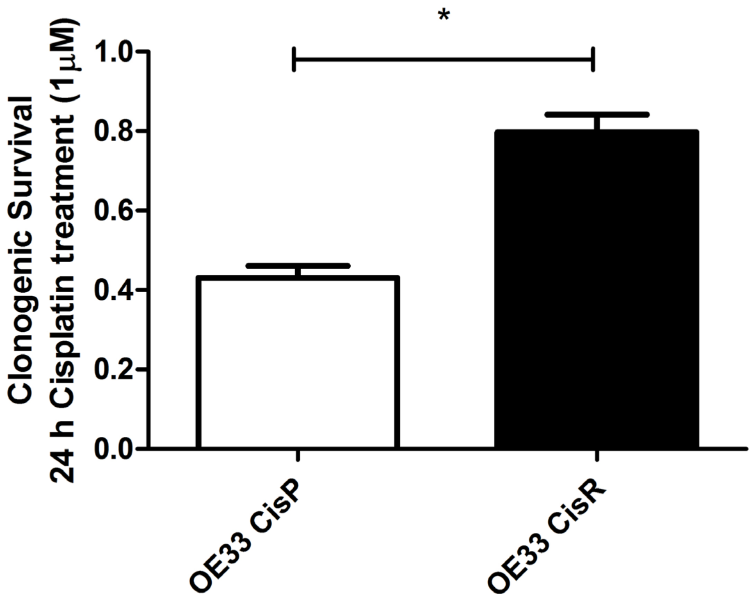

Supplementary Figure 1: Clonogenic survival was assessed in OE33 CisP and OE33 CisR cell lines following 24 h treatment with cisplatin (1 μM). OE33 CisR cells demonstrated significant resistance to cisplatin, when compared to OE33 CisP cells. Data are presented as mean ± SEM from 3 independent experiments. Statistical analysis was performed using a two-tailed paired Student's *t*-test, $*p < 0.05$.

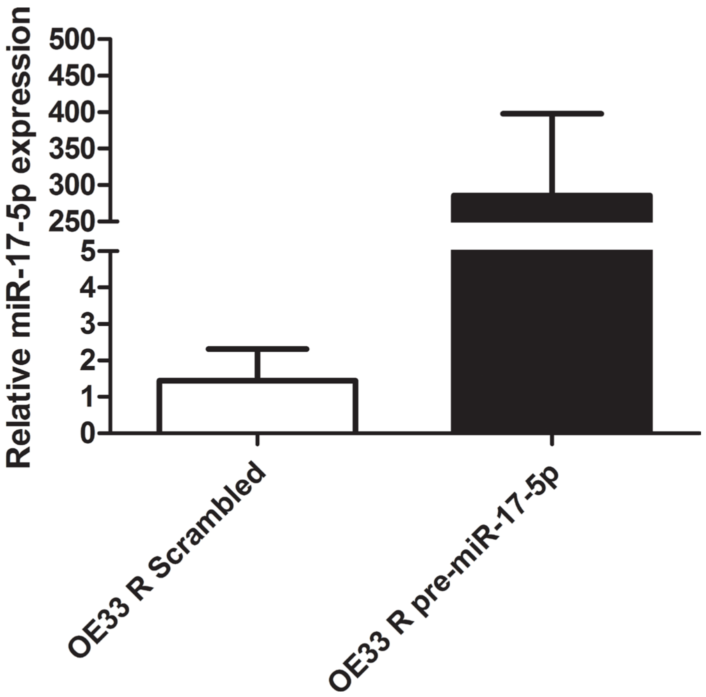

Supplementary Figure 2: miR-17-5p expression was measured by qPCR in OE33 R cells transfected with either pre-miR-17-5p or a scrambled non targeting control.
